# The Role of Protein Interactions in Mediating Essentiality and Synthetic Lethality

**DOI:** 10.1371/journal.pone.0062866

**Published:** 2013-04-29

**Authors:** David Talavera, David L. Robertson, Simon C. Lovell

**Affiliations:** Faculty of Life Sciences, University of Manchester, Manchester, United Kingdom; Hospital for Sick Children, Canada

## Abstract

Genes are characterized as essential if their knockout is associated with a lethal phenotype, and these “essential genes” play a central role in biological function. In addition, some genes are only essential when deleted in pairs, a phenomenon known as synthetic lethality. Here we consider genes displaying synthetic lethality as “essential pairs” of genes, and analyze the properties of yeast essential genes and synthetic lethal pairs together. As gene duplication initially produces an identical pair or sets of genes, it is often invoked as an explanation for synthetic lethality. However, we find that duplication explains only a minority of cases of synthetic lethality. Similarly, disruption of metabolic pathways leads to relatively few examples of synthetic lethality. By contrast, the vast majority of synthetic lethal gene pairs code for proteins with related functions that share interaction partners. We also find that essential genes and synthetic lethal pairs cluster in the protein-protein interaction network. These results suggest that synthetic lethality is strongly dependent on the formation of protein-protein interactions. Compensation by duplicates does not usually occur mainly because the genes involved are recent duplicates, but is more commonly due to functional similarity that permits preservation of essential protein complexes. This unified view, combining genes that are individually essential with those that form essential pairs, suggests that essentiality is a feature of physical interactions between proteins protein-protein interactions, rather than being inherent in gene and protein products themselves.

## Introduction

In all organisms there is a complex relationship between genotype and phenotype. A key tool in genetic research is the generation and analysis of null mutations, whereby a particular gene is rendered non-functional, often through deletion. Analysis of null mutants allows the identification of “essential genes”, which are inferred to have the most significant contributions to function in a given environment.

In yeast (*Saccharomyces cerevisiae)* only around 20% of genes are essential when grown in the laboratory [1]. Many factors determine whether a given gene is classed as essential: essentiality has been linked with its protein product being highly connected in networks of protein-protein interactions [2], although this so-called “centrality-lethality rule” has been disputed [3]. Alternatively, genes can be essential if the proteins they code for are involved in specific biological modules such as protein complexes [4] or if they participate in a subset of interactions that are themselves essential [5]. Nevertheless, it is clear that the essential nature (or otherwise) of a gene is strongly dependent on context and environmental conditions [6], [7].

**Figure 1:**
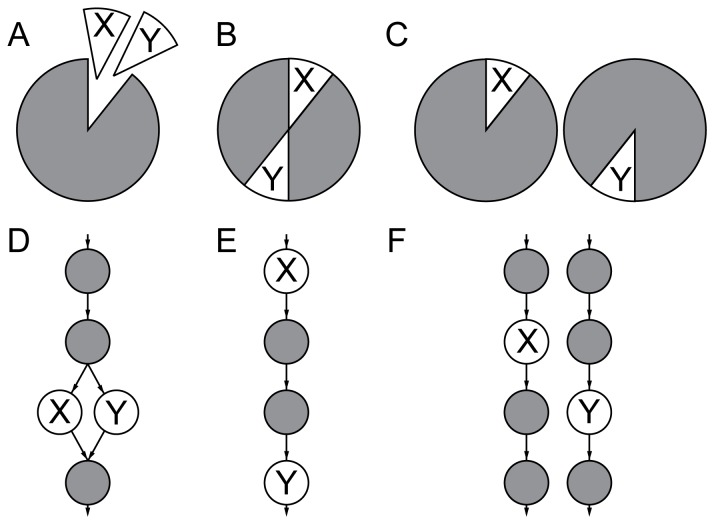
Potential mechanisms that can give rise to synthetic lethality (essential pairs). X and Y indicate proteins that are coded for by synthetic-lethal gene pairs. A–C gray indicates other protein complexes; D–E gray circles indicate other proteins that are part of a pathway. A. Proteins X and Y are functionally redundant members of a multi-protein complex. B. Proteins X and Y are both members of the same protein complex. C. Proteins X and Y are essential members of two different protein complexes that can functionally compensate each other. D. Proteins X and Y are redundant members of a metabolic pathway. E. Proteins X and Y play a synergistic role in a metabolic pathway. F. Proteins X and F are essential members of alternate metabolic pathways.

An important aspect of the context in which a gene functions is the genetic background in which it is expressed [8]. Genetic interactions are common [9], and arise when simultaneous mutation of more than one gene results in phenotypic effects greater or less than the multiplicative effects of mutating each gene individually. The most extreme case of negative genetic interaction is synthetic-lethality, where genes are not essential when mutated or deleted individually, but are lethal when altered simultaneously. This phenomenon can be understood through a buffering effect [10]: gene *X* can buffer the phenotypic effect of the loss of gene *Y* and vice versa. However, if both are lost simultaneously no buffering is possible. Alternatively, there may be a less direct, additive effect, where each deletion causes a decrease in the ability of a pathway to function. A number of mechanisms involving the protein products can explain how such buffering may come about (Figure 1), including some previously proposed [10]. Using the nomenclature of Kelley and Ideker [11], A, B, D and E are “within pathway” explanations of genetic interactions, whereas C and F are “between pathway” explanations. The terms “within module” and “between module” have also been used for similar concepts [12].

Protein-protein interactions can account for a relatively small proportion of genetic interactions [9]. Indeed, protein complexes seem to mostly account for subsets of monochromatic genetic interactions (mostly positive or mostly negative, but not equally mixed) grouped into functional modules [12]. Interestingly, one recent study has related the strength of the genetic interaction (either positive or negative) with the proportion of common physical interactors [13]. Similarly, genetic interactions have been rationalized using metabolic models [14]. Although there are discrepancies between predicted genetic interactions and those measured experimentally, pleiotropy can often be explained through the use of such models [14]. An alternative hypothesis relates synthetic lethality to rearrangements of the genetic interaction network after one gene is deleted [15]; however, since we do not understand how such rearrangements occur, it is not possible to predict the outcome of novel genetic interactions.

A key aspect of genetic context of a gene is the presence of a duplicate copy. Gene duplication initially produces two or more identical genes that may diverge in function [16]. These duplicated genes may functionally compensate for each other [17], [18], even up-regulating the expression of the remaining gene if needed [19]. More than 30% of yeast duplicate genes are functionally redundant, and this is the case whether they arise from whole genome duplication or smaller scale duplications [16], [20]. Moreover, there is little evidence for functional redundancy between unrelated singleton genes. Thus, genes that have not been duplicated are more likely to be essential than those that have duplicated [17], [21], [22]. However, this is not true in mammals [21], [23]–[25], probably due to the complexity of development of higher organisms [26], [27].

Duplicates that have a high degree of sequence similarity are more likely to be able to functionally substitute for each other [28]. Conversely, genes that have not been duplicated or genes where the duplicate pairs have a high degree of sequence divergence are unlikely to be able to compensate for each other [22], [29]. Although the maintenance of highly-similar paralogs could be detrimental [30], there are examples where network redundancy can ensure a high metabolic flux [6] or provide a rapid response to changing conditions. An example of the latter is the regulation of gene expression by transcription factors [31].

There have been several estimates of the percentage of negative genetic interactions that were caused by the simultaneous deletion of duplicate genes [18], [32]; however, the estimates greatly differ. Some differences could be due to the size of the dataset of negative interactions, or to the methodology for finding duplicates. As no study has been exclusively devoted to synthetic lethal pairs, we do not know the proportions for this particular set of negative genetic interactions. For instance, it is unknown whether synthetic lethality (and synthetic sickness) are regulated through the same mechanisms, or have different origins, e.g. one set being Mendelian traits and the other being quantitative traits.

Of the basic potential mechanisms by which synthetic lethality can arise (Figure 1), mechanisms A, C, D and F can clearly arise through gene duplication. In mechanism A, proteins X and Y, coded for by a synthetic lethal pair of genes *X* and *Y*, functionally compensate for each other by binding to their partner through the same interface. If *X* and *Y* were recently duplicated paralogs, their protein products would be expected to have similar binding specificities, and so be able to compensate in this way. Similarly, in mechanism D, proteins X and Y carry out the same step in a metabolic or signaling pathway. Mechanisms C and F may arise through duplication of the whole subsystem. In mechanism C, the entire complexes of which X and Y are members may be functionally redundant, and may be paralogous. Duplication of whole complexes must happen in concert for them to remain functional; this can be achieved through whole-genome duplication [33]. Similarly mechanism F may arise through duplication of a whole subsection of a pathway. Alternatively for both mechanisms C and F there may be an alternative independent mechanism to produce the same function. In the latter case functional redundancy may arise without the members of the complexes or pathways being paralogous. Obviously, these basic mechanisms can be further complicated; e.g.: three parallel pathways, of which any two must be present.

Many proteins pairs that are coded for by synthetic lethal genes participate in protein complexes [34], and synthetic lethality may arise when paralogs compensate for each other within complexes (Figure 1A) [35]. However, several studies of the relationship between genetic interactions and physical interaction networks have concluded that the majority of genetic interactions occur between proteins in different complexes [11], [36], suggesting minimal functional compensation. Interestingly, a genome-wide association study reached the same conclusions [37]. Mapping the majority of genetic interactions in yeast demonstrates 1) that there is only modest overlap between genetic interactions and direct physical interactions and 2) that proteins with similar functions have a similar pattern of genetic interactions [38]. Exceptions include those sets of interactions termed “monochromatic” (*i.e.*, predominantly positive or predominantly negative); modules defined in this way are frequently associated with protein complexes, although they account for only a minority of cases [12]. Two studies have demonstrated that most protein complexes with only negative interactions also have components coded for by essential genes [39], [40]. Surprisingly, these studies included genetic interactions with essential genes carrying hypomorphic mutations, which may affect the results as those proteins are at the same time part of the criterion for analyzing the data (complexes with monochromatic negative genetic interactions), and the object of study.

Here, we present a study of the relationship between essential genes and synthetic lethal pairs in yeast, which we consider having paired essentiality. We restrict the analysis to synthetic lethal pairs, and differentiate these from other negative genetic interactions, an approach that differs from previous studies [12], [39], [40]. We demonstrate that 1) the presence of duplicate copies is not the major determinant of whether a gene is essential and, 2) products of essential genes and members of synthetic lethal pairs frequently interact with each other. We expand the analysis of He and Zhang [5] to include both essential genes and essential pairs. We therefore present a new view of essentiality, emphasizing the importance of protein complexes. This view focuses on protein-protein interactions, rather than on the proteins themselves.

## Results

### Gene Duplication does not Explain Synthetic Lethality

Presence or absence of duplicate genes has been used to explain the existence of synthetic lethal pairs and essential genes, respectively. If duplicate genes are the main explanation we would expect 1) that nearly all essential genes to be singletons (*i.e.,* without a paralog); and, 2) that most synthetic lethal pairs to be paralogous. Importantly, we would not expect that all gene duplications give rise to a synthetic lethal pair [18].

**Figure 2:**
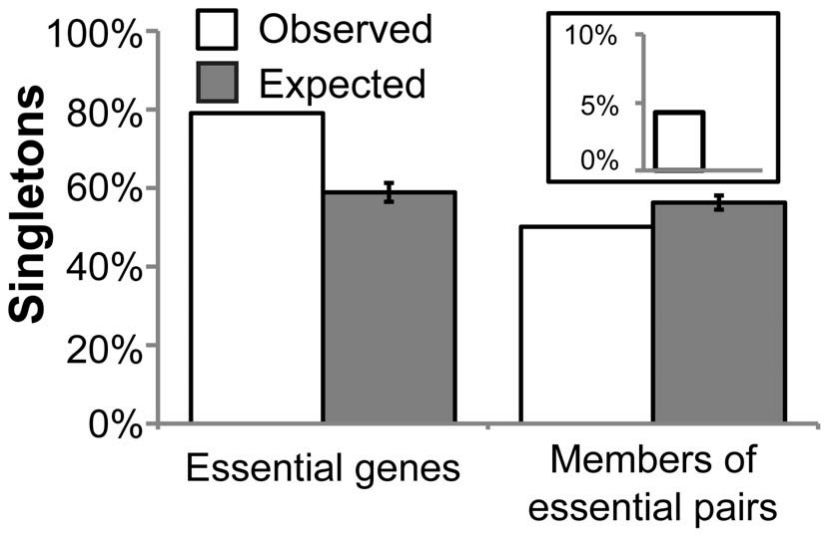
Duplication of essential genes and members of essential pairs. Genes were selected using stringent criteria. The inset shows the percentage of essential gene pairs that are paralogs.

We find that in yeast 79.1% of essential genes selected using the stringent criteria are singletons (78.5% for those selected using the tolerant criteria, see methods section for selection criteria; Figure 2 and Table S1). This is much higher than expected by chance (58,9%; p-value <10^−4^). By contrast, only 50.1% of the genes in synthetic lethal pairs selected using the stringent criterion are singletons (49.5% for those selected using the tolerant criteria), which is lower than expected (56.3%; p-value = 3×10^−4^). These results agree with previous research, indicating that single-gene-associated essentiality derives from a lack of paralogs [21], and that synthetic lethality originates by gene duplication [22], [29], [35], [41]. However, as with the complete set of negative interactions [17], [18], [32], this is not a complete explanation. First, more than 20% of yeast essential genes have paralogs in the yeast genome. Second, half of the genes in synthetic lethal pairs do not have any paralog. Third, although half of the genes that are members of synthetic lethal pairs have paralogs, the percentage of synthetic lethal pairs where the members are paralogous genes is just 4.3% (2.5%, when using the tolerant criteria). This percentage is more similar to that originally found for the whole set of quantitative negative interactions [32] than to more recent estimates [17], [18].

Some of the paralogs of essential genes might correspond to highly diverged duplicates or paralogs with substitution of functionally important residues. In both cases, modifications might prevent functional compensation [16], [28]. In the case of synthetic lethal gene pairs, it is unlikely that most of the pairs were duplicates that are able to compensate functionally, but have diverged so much to prevent their identification as paralogs. It is therefore highly probable that mechanisms other than duplication events must contribute to determining whether or not a gene is essential.

Some synthetic lethal pairs might not participate in functional redundancy through duplication. Rather they may contribute to robustness due to the existence of alternative pathways or complexes that perform the same function but are not paralogs [10], [22]. Alternatively, both members of the essential pair could be present in the same complex or metabolic pathway [10], adding a small but synergistic effect (see Figure 1B&E).

### Members of Synthetic Lethal Pairs are Functionally Related

**Figure 3:**
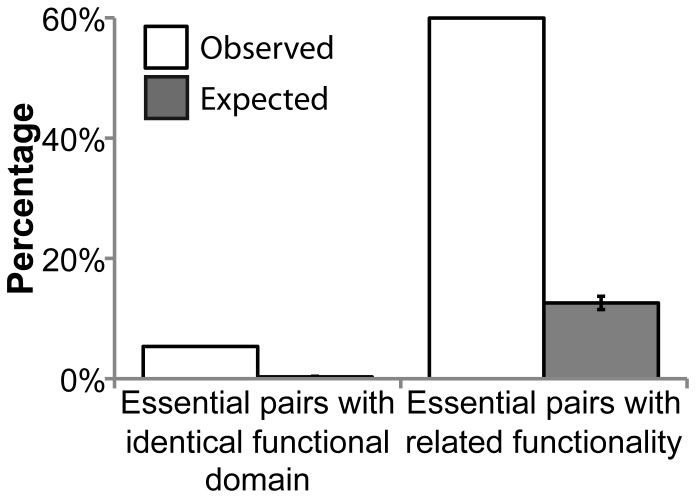
Evolutionary and functional similarity of essential pairs. Genes selected using stringent criteria. For the expected values the mean and standard deviation are shown.

Synthetic-lethal pairs that are able to compensate for each other through physical substitution are, by definition, interchangeable, and are therefore functionally related. Valente and co-workers [35] found several examples of paralogs that are likely to be interchangeable in both obligate and transient complexes. In agreement with the low percentage of synthetic lethal pairs that are paralogs (Figure 2 and Table S1), we find that only 5.4% (3.6% using the tolerant criterion) of the protein products of the essential gene pairs have at least one functional domain, as defined by InterPro, in common (Figure 3 and Table S2). Even though this percentage is higher than the random expectation, it confirms that a small minority of synthetic lethal pairs are generated by simultaneous knockout of evolutionary-related genes.

By contrast, the disruption of related functions seems to explain a large proportion of synthetic lethality, as was demonstrated in one of the first studies using synthetic genetic arrays [42]. We identified functions of each gene and compared these assignments between members of synthetic lethal pairs. More often than expected by chance (60.0% vs. 12.6% when using the stringent criterion; p-value <10^−4^) synthetic lethality involves the deletion of genes working in the same or related functions (Figure 3 and Table S2). This indicates that many examples of synthetic lethality are caused when a single functional process is disrupted, either by the deletion of two redundant proteins, or disruption of alternate pathways. Our figures are higher than those of Tong and coworkers [32]; however the results are difficult to compare because they consider synthetic sick pairs as well as synthetic lethal pairs, whereas we restrict our analysis to synthetic lethality. It could be that functional compensation was mainly related to synthetic lethality, with synthetic sick interactions emerging due to different causes (Table S3). However, the differences in sample size and the ambiguity of the definition of both genetic interactions suggest caution in interpreting this result. There is the possibility of “study bias”, whereby genes selected for experimental testing are likely to be functionally related. This bias for functional relatedness could be introduced 1) when performing small-scale experiments (in 323 out of 440 sources reporting just one synthetic lethal pair, that pair is functionally related); or, 2) when using epistatic miniarray profiles (E-MAP), which are designed for testing only a subset of interactions suspected to be enriched for positive or negative interactions [43], [44]. The majority of data in our data set came from experiments not using functional information in their design; nevertheless even these subsets displayed a degree of functional relatedness (Table S4).

### Most Synthetic Lethal Pairs are Caused by the Deletion of Members of Protein Complexes

Synthetic lethality due to functional redundancy can occur if two genes code for proteins that buffer each other. Thus, being in protein complexes or acting in metabolic pathways, the deletion of one gene is buffered by the other’s presence (Figure 1); a wider explanation on the causes of epistasis, of which synthetic lethality is the ultimate consequence, was given by Lehner [45]. In order to determine whether membership of a protein complex or participation in a pathway are equally important for understanding synthetic lethality, we calculated the percentage of synthetic lethal pairs where both members were assigned to a metabolic pathway or were detected by affinity capture experiments. We assume that most affinity capture experiments retrieve multi-protein complexes. Thus we assign a status as member of a protein complex to any protein involved in protein-protein interactions detected through affinity capture experiments. Our results show that 89±1% of the synthetic lethal pairs contain two genes that code for such proteins, suggesting that gene products being members of the same or different protein complexes is one of the main reasons for synthetic lethality. As some proteins could be found as hits in affinity capture experiments despite not being involved in protein complexes, we also used an alternative and more restrictive definition of complex membership, based on GO terms. Although the percentage of synthetic lethal pairs where both genes were members of protein complexes was lower, under this definition we still find that the figures are 40±0% if using all annotations but for the IEA, NAS and ND ones, or 38±0% if only using annotations with an experimental evidence code. Conversely, the disruption of metabolic pathways can explain only a minority (12±0%) of cases of synthetic lethality. Moreover, only 22±5% of the synthetic lethal pairs caused by the metabolic factor occur in the same pathway, suggesting that alternate pathways account for ∼10% of synthetic lethal pairs.

**Figure 4:**
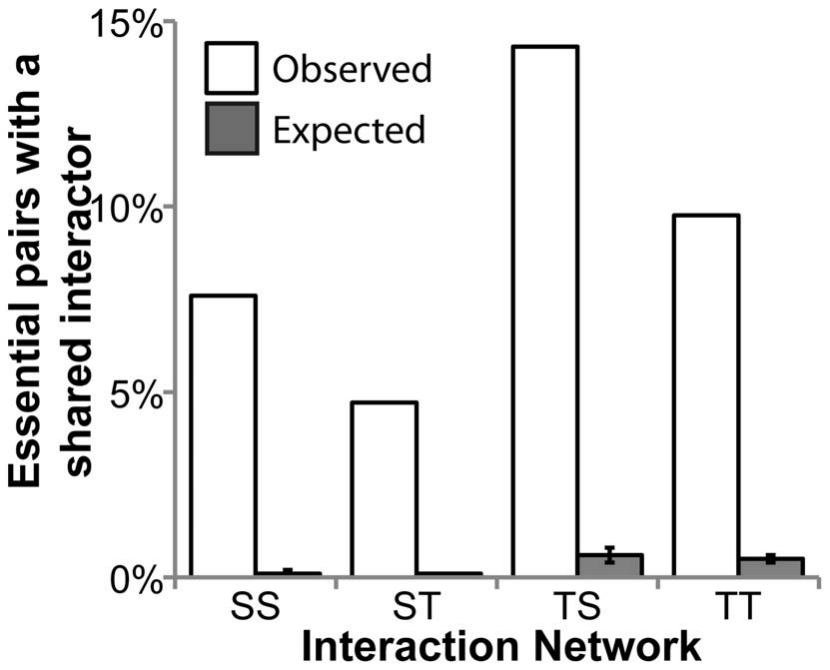
Essential pairs where both members bind the same interactor. The four interaction networks are built combining the two criteria for selection of essentiality and physical interactions (SS if two stringent criteria were used; ST if stringent criteria was only used for selecting physical interactions; TS if only essentiality was stringently selected; and TT if only tolerant criteria were used). For the expected values the mean and standard deviation are shown.

Synthetic-lethal pairs with gene products in different complexes are more common than pairs in the same complex [11], [36], although there may be methodological or statistical biases [11], [36]. Nevertheless, there are several examples where products of synthetic lethal pairs are present in the same protein complex [11], [34], [36], even if they are not expected to directly interact [9]. Indeed, if proteins have a compensatory role we expect that they will not interact with each other; rather they should have interacting partners in common [13]. This is the case for many synthetic lethal pairs (Figure 4 and Table S5). The observed percentages are much higher than those obtained by chance (7.6% vs. 0.1–0.7% when analyzing the SS network; p-value <10^−4^) and are independent of the structure of the synthetic lethality network, since altering the network topology and substituting or reshuffling nodes produce a significant decrease in the number of synthetic lethal pairs with a common physical interactor. Most importantly, for 63±4% of pairs with an interacting partner in common, the shared interactor is either the product of an essential gene or a member of a synthetic lethal pair. Increase in the number of experimentally-identified protein-protein interactions can only increase the number of pairs sharing interactors. These findings indicate that a number of synthetic lethal pairs code for putatively interchangeable proteins that form protein complexes. In this case the simultaneous knockout of both proteins produces a lethal phenotype because the complexes cannot be formed.

### “Essential Proteins” Group into an Interaction Sub-network

The importance of protein complexes for synthetic lethality indicates that the formation of these protein complexes is crucial for the survival of the cell. This implies that the other components of these complexes must also be essential [39]. Accordingly, we hypothesize that “essential proteins”, regardless of whether they are the product of essential genes or members of synthetic lethal pairs, should be highly interconnected. This is an expansion of the original essential interactions hypothesis proposed by He and Zhang [5] and is supported by the previous finding that some protein complexes are enriched with single-gene essential proteins and products of synthetic lethal pairs [34].

In order to verify the expected enrichment on interactions between essential proteins, we determined the number of essential proteins that physically interact with other essential proteins. As a control, we calculated the percentage of non-essential proteins interacting with either single-gene essential proteins or members of synthetic pairs. We find that 75±9% of the single-gene essential proteins and 81±5% of members of synthetic lethal pairs interact with other essential proteins, whereas just 57±10% of the non-essential proteins interact with one or more essential proteins. This may be because products coded for by essential genes tend to be highly connected.

**Figure 5:**
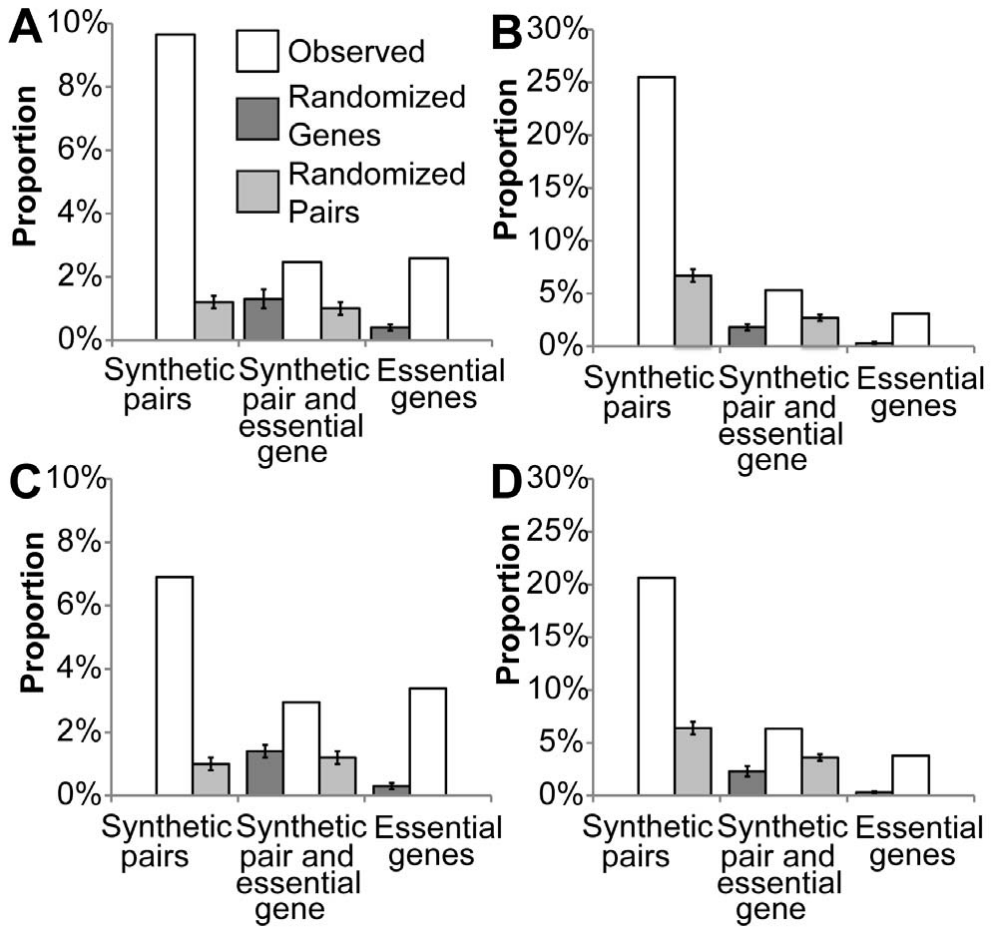
Essential interactions. A. SS network. B. ST network. C. TS network. D. TT network. “Randomized genes” indicates the mean and standard deviation of expected values with random assignments of essential genes. “Randomize pairs” indicates the mean and standard deviation of expected values with random assignments of essential pairs.

**Figure 6:**
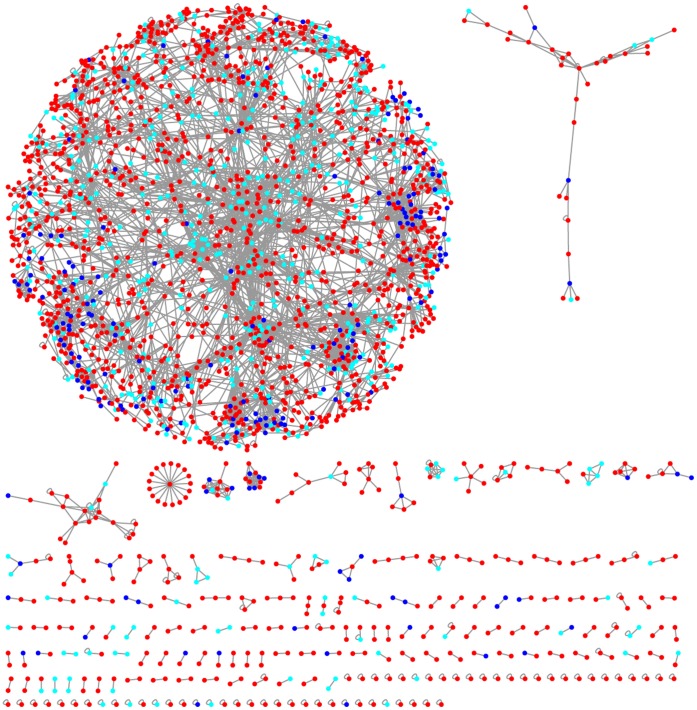
Biggest component of the physical interaction network. Proteins coded by essential gens are coloured in dark blue; proteins that are members of synthetic lethal pairs are coloured in light blue; and, non-essential proteins are coloured in red.

We also determined the number of interactions between essential proteins, comparing the results with the networks annotated using purely randomized lists of interactions. Annotation is independent of the synthetic lethality network (i.e. degree distribution), and only takes into account whether a gene is involved in any synthetic lethality interaction. First, we determined the frequency by which essential-gene proteins interact with each other. Although just 2.6%–3.8% of physical interactions occur between the products of two essential genes, these results are higher than expected by chance (0.3–0.4%; p-value <10^−4^; Figure 5 and Table S6). We find, therefore, that single-gene essential proteins tend to interact with each other [5], [46], meaning that they not only cluster in functional modules [4], but also tend to be in the same complexes [34], [47]. In addition, the number of physical interactions involving members of synthetic lethal pairs is higher than that expected by chance (Figure 5 and Table S7). Finally, we also observe an enrichment of interactions between essential genes and members of synthetic lethal pairs (Figure 5 and Table S8). Since this enrichment is independent of the dataset randomisation used, we conclude that these interactions are not caused by the distribution of just one type of essentiality. Importantly, removing physical interactions detected through affinity capture methods causes only a small, non-significant decrease of the proportion of physical interactions involving essential genes or members of synthetic lethal pairs (Table S9). These results corroborate our essential interactions hypothesis and demonstrate that essential proteins are highly inter-connected, and so essential proteins define an interaction sub-network (Figure 6). Although apparently members of synthetic lethal pairs are more likely to self-interact (Table S10), this is probably a by-product of the different combination rules for hetero- and homo-complexes. Indeed, the essential interaction sub-network is mainly formed by hetero-interactions (Table S10).

Finally, we determined whether there were differences between networks exclusively composed of obligate or transient protein interactions. These two types of protein interactions are quite disparate [48]; so, potentially, they could introduce differences on the network. Although it is not clear if there are differences in the proportion of interactions involved in the essential subnetwork (Table S11), it seems clear that physical interactions between the products of essential genes is more relevant for obligate interactions (Table S12), whereas transient interactions contain a greater percentage of interactions between the proteins coded by genes involved in synthetic lethality (Table S13). These differences are due to the fact that the proportion of products of single-gene essentiality is much higher in the networks of obligate physical interactions (Table S14). Nonetheless, we prefer to be cautious about these results, as they could be caused by our definition of obligate and transient interactions (see Methods).

### Functional Convergence may Diminish the Number of Essential Genes

**Figure 7:**
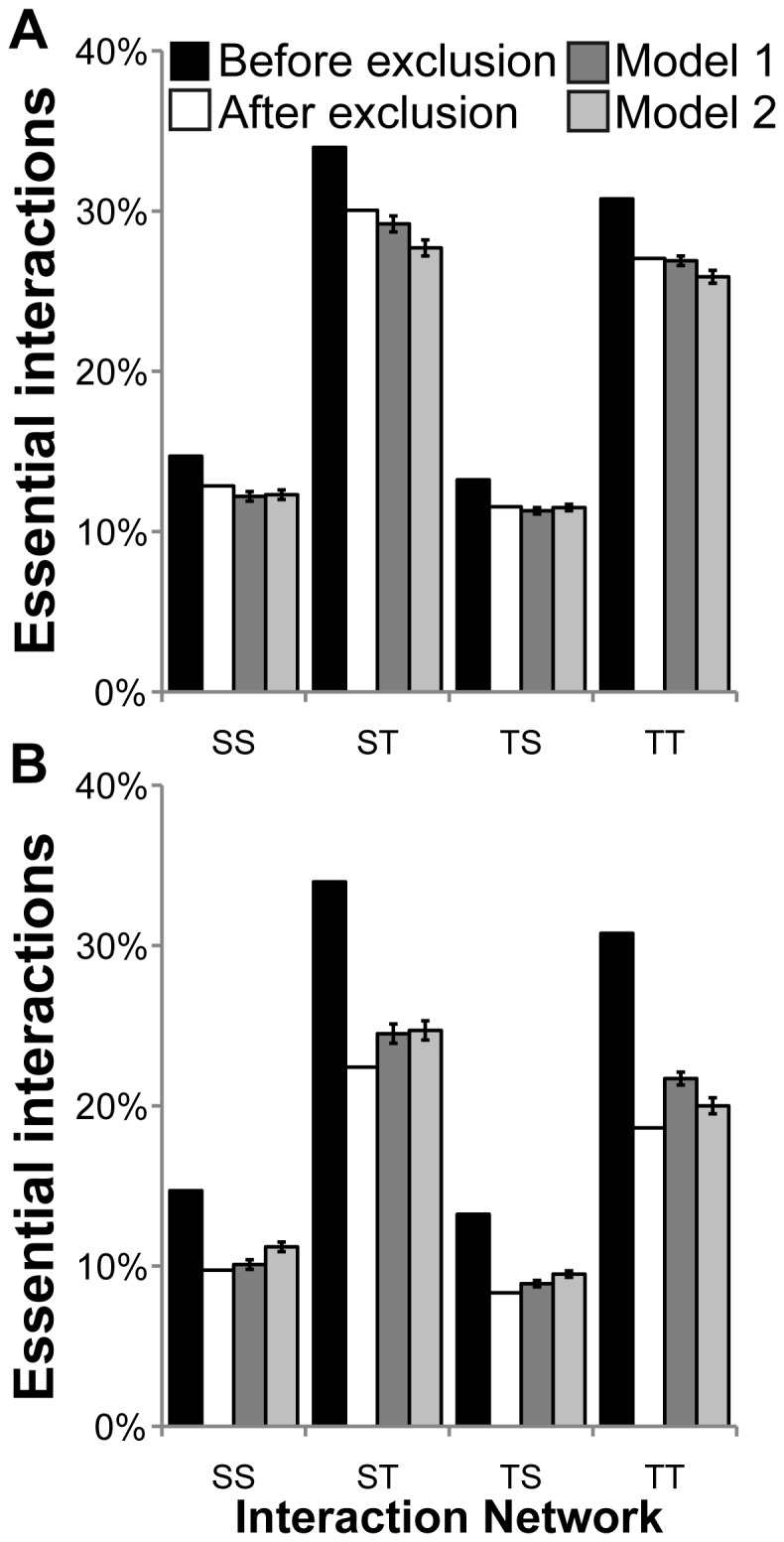
Contributors to essential interactions. A. Exclusion of essential pairs where genes are paralogs. B. Exclusion of essential pairs where proteins share interactors. “Before exclusion” original values before exclusion of essential pairs; in blue; “After exclusion” observed values after the exclusion of selected pairs; “Model 1″ mean and standard deviation of expected values based on model 1 (exclusion based on number of genes); “Model 2″ mean and standard deviation of expected values based on model 2 (exclusion based on number of pairs).

Clearly, randomly removing essential proteins from the analysis should diminish the enrichment in the essential sub-network. We separately excluded (a) members of synthetic lethal pairs that were paralogs and (b) members of synthetic lethal pairs that shared a common interacting partner. However, although the effect of the exclusion of paralogs is as expected, exclusion of pairs with an interacting partner in common has a greater effect (Figure 7 and Table S15).

These results demonstrate that the ability to form the correct, cognate interaction leads to the enrichment of physical interactions between “essential proteins”, rather than any evolutionary relationship. Moreover, there may be functional convergence so as to preserve essential protein complexes. We speculate that some of the singletons that are members of synthetic lethal pairs could have become non-essential through this buffering mechanism.

## Discussion

It has been suggested that for relatively simple organisms, such as yeast, the phenomenon of gene essentiality may be easily related to duplication [21]: singleton genes (*i.e.,* those without a clear paralog) are likely to be essential, whereas duplicated genes may compensate for each other [28]. Our results show that even in yeast there is no straightforward relationship between essentiality and gene duplication.

We find that one fifth of essential yeast genes have paralogs; however, when synthetic lethal pairs are considered, in only 4.3% of cases are the two members of the pair paralogous. This figure is lower than some estimates of weak phenotypic effect due to duplicate redundancy [17], [18], and is similar to that of an older study that demonstrated that just 2% of all negative genetic interactions involved duplicate genes [32], notwithstanding methodological differences. Nevertheless, it is clear that, even in yeast, synthetic lethality cannot be explained by duplication alone.

Perhaps unsurprisingly, the ability for synthetic lethal gene pairs to compensate functionally is much more important. This is the case whether they are divergently or convergently evolved. We find that this functional compensation is highly likely to be due to membership of the same protein complex, and is frequently due to both members of a synthetic lethal pair sharing a common interacting partner. Thus, the most common type of functional compensation arises if two proteins are able to compensate for each other’s protein-protein interactions, rather than by any other mechanism (Figure 1). Conversely, genes often have the property of single-essentiality if their protein products make interactions that cannot be compensated. The key observations are, therefore (i) that the clustering of essential genes and synthetic lethal pairs in the protein interaction network suggests that synthetic lethality is strongly dependent on the formation of protein-protein interactions and (ii) that compensation most frequently arises due to functional similarity that results in the preservation of essential protein complexes. The central role of the interactions in synthetic lethality allows an alternative phrasing: namely that it is the interaction that is essential, rather than the gene.

If our proposed interaction-centric view is correct, can help understanding human disease [49]. Here we have applied it to the understanding of essentiality, extending the of work of He and Zhang [5]. An interaction focus allows us to unify the analysis of essential genes, synthetic lethality and functional compensation within a single framework. We propose that this single framework helps to overcome the contradictions described by other researchers [4].

Previous research had shown the presence of the products of essential genes and genes with negative genetic interactions in the same complex [39], [40]. Here, we demonstrate enrichment for physical interactions of a subset of these products: i.e. we find that there is an interaction sub-network that physically links functionally-related “essential proteins”. This essential sub-network becomes apparent when essential genes and genes with paired-essentiality (synthetic lethal pairs) are considered together. Although cautiously, our results suggests that the macromolecular machinery would make most of the physical interactions involving the protein products of single-essential genes, whereas the members of synthetic lethal pairs might have a relatively more important role on transient interactions. Previous research found that essential interactions are more evolutionary-conserved than non-essential interactions [5], [50]. This is also true for a significant part of the genetic interaction network [51]. It is possible that this set of interactions represents the ancestral network, and is the core set of functions that are common to a wide range of organisms. We therefore expect that most of the essential sub-network will be found in related organisms. Interestingly, research on bacterial metabolic networks shows that the network core is the most conserved, whereas the peripheral interactions originated by niche specialization of the different organisms [52].

Since compensation does not mainly occur because of an evolutionary relationship, removing paralogous synthetic lethal pairs from the network analysis has no greater effect on the essential sub-network than removing random essential pairs. By contrast, a higher depletion of essential interactions is observed when removing pairs that have at least one interacting partner in common. Thus functional similarity, most commonly manifested as similarity of protein-protein interactions, permits preservation of essential protein complexes. Consequently, in many cases, lethality is due to the lack of an essential component of the protein complex. These results highlight the protein complex as the basic essential functional unit [12], with the specificity of the protein-protein interaction being the key to compensation.

Due to the expected conservation of interactions involving essential proteins [5], [50], [51], it is likely that the definition of a comprehensive essential interaction sub-network may aid in the prediction of many protein-protein interactions in related organisms. Experimentally-generated genetic interactions can give a mechanistic explanation of the phenotype associated with a range of naturally-occurring mutations [37]. Moreover, protein complexes not only form functional units, but also play a central role in linking other functional units [12]. There may therefore be great benefit for biotechnology and medicine in studying the consequences of protein mutations associated with the disruption of essential interactions, rather than the protein itself. Further, we hypothesize that this interaction-centric view of essentiality is likely to facilitate understanding of essentiality in mammals.

## Methods

### Data

The complete list of physical and synthetic lethal interactions data was downloaded from the BIOGRID repository (http://thebiogrid.org/; version 2.0.63 downloaded on 25^th^ March 2010) [53]. Essential genes were recovered from different sources: 1083 genes from the *Saccharomyces* Genome Deletion Project [1], [54]; 1007 genes from the CYGD database (http://mips.helmholtz-muenchen.de/genre/proj/yeast/) [55]; 1136 genes from the SGD database (http://www.yeastgenome.org/) [56] and 1107 genes from a recent study using synthetic genetic array technology [9]. In order to keep a proper balance between the quality and the size of the dataset, we used two different criteria to select each type of data. Paralogs and InterPro domains assignments were downloaded from Ensembl Genomes [57] via the BioMart portal [58]. Functional clusters were extracted from the DAVID bioinformatics resources (http://david.abcc.ncifcrf.gov/) [59], using the highest stringency and setting the initial and final group membership to 2.

### Physical Interactions

In the case of the physical interactions, we encountered two problems: (1) the methodology used for detecting the interaction, and (2) the reproducibility of the results. Different methodologies account for different types of detected interactions, each with strengths and weaknesses. For instance, yeast-two-hybrid experiments identify whether two proteins bind each other; however, biologically irrelevant interactions can also be detected. Affinity capture methods are believed to account for many false positives, whereas they allow detection of interactions without the need for protein over-expression. The relative merits have been discussed previously [60]–[62]. Moreover, Yu and coworkers found that although yeast-two-hybrid technique was better for finding direct interactions than affinity purification methodologies, they were more prone to be biased towards essential genes or genes encoding specific functions [46].

As all methods have their own sources of errors, we (1) selected those detected using two different experimental systems and reported in two different scientific papers (2350 nodes, 4951 edges, stringent criteria) and, (2) selected those with two different pieces of evidence, *i.e.,* being reported twice or being detected using two different methods (3335 nodes, 12153 edges, tolerant criteria)(see Figure S1 and Tables S16 and S17 for details). Most of the physical interactions selected using the stringent criterion are likely to be true positives in terms of the protein’s ability to bind and in their biological relevance. However, we can not rule out that some of them are artifacts identified using different experimental designs.

### Essential Genes and Synthetic Lethal Pairs

In order to clearly differentiate essential genes and genes in synthetic-lethal pairs, those essential genes that are also members of synthetic-lethal pairs were removed from the datasets. Sometimes the genetic interactions are measured between a non-essential gene and an essential gene containing a hypomorphic mutation. Although these interactions are biologically relevant, they can be harder to interpret. Moreover, it is not logically correct to classify a gene as being essential and non-essential simultaneously. A possible consequence of using the whole data would be that results for members of synthetic-lethal pairs would be heavily influenced by results for essential genes. Thus, in contrast to previous research [39], [40], we removed these genes from our analyses. Another problem we encountered is that genetic interactions are not comprehensively tested, even in high-throughput studies. Experiments may therefore focus on specific sets of genes. In addition, high-throughput experiments are limited to a small number of research groups. Nevertheless we believe that the numbers of synthetic-lethal interactions we have analyzed are a fair representation of the current knowledge.

Data set selection is illustrated in Figure S2, and described in Tables S18 and S19. When using the stringent criterion, we selected the synthetic pairs reported twice independently (694 nodes, 1621 edges) and the essential genes present in all the four datasets (387 nodes). When using the tolerant criterion, we selected all the synthetic-lethal pairs (2007 nodes, 8055 edges) and the essential proteins reported in at least three datasets (437 nodes). Although some synthetic-lethal interactions could be reported twice for the same research group, and the possible existence of an ambiguity between synthetic-lethality and synthetic-sickness, the variety of sources (see Tables S18 and S19) make us confident that most of synthetic-lethal interactions selected using the stringent criteria are true positives. Nevertheless, we built two additional control lists: one of high-confident synthetic-lethal pairs, and the other of possible synthetic sick pairs. In order to generate a set of pairs with small chance of containing false positives, we explored the list generated with the stringent criteria, and only selected 113 pairs that were reported in at least four different publications. The fact that there are 92 different sets of publications reassures that there is small chance of a reporting bias. For generating a list of synthetic sick pairs, we selected all pairs of genes involved in genetic interactions termed “Negative Genetic” or “Synthetic Growth Defect”. Then, we discarded those pairs that had been identified participating in a synthetic lethal interaction, and those pairs containing a gene identified as essential in any of the 4 datasets previously mentioned. This resulted in a list of 53902 pairs of putative synthetic sick pairs.

### Protein Complexes

We used two different definitions of protein complex. In one, we defined a protein as a member of a protein complex if it had been detected participating in protein interactions by means of affinity capture methods. In the other, we used a similar strategy as Michaut and coworkers [12]: we considered all genes annotated with the GO term macromolecular complex (GO:0032991) and its children terms, excluding the annotations with the qualifiers “NOT” and “colocalizes_with”. We generated two different sets of proteins: 1) those for which there was experimental evidence for the annotation; and, 2) those annotated by any means except when there was a non-traceable author statement (NAS evidence code), no biological data available (ND evidence code) or inferred from electronic annotation (IEA evidence code).

### Obligate Transient Interactions

We divided our set of physical interactions in two different subsets: obligate and transient interactions. Unambiguous differentiation of these two interaction types can be challenging since there is a continuum of binding affinity [48]. Here, we classified as obligate interactions those detected through affinity capture experiments and involving proteins annotated as participating in macromolecular complexes (GO:0032991) through experimental evidence. Conversely, transient interactions included those detected by any other method than affinity capture and involving only proteins that were not assigned to participate in protein complexes. It is noteworthy that 1) some of the interactions could not be assigned to any of the subsets; and, 2) the way we built the networks does not allow crosstalk between proteins in obligate interactions and proteins in transient interactions.

### Annotated Networks of Physical Interactions

Combining the selection criteria, we built four networks of physical interactions annotated with essentiality information (see Figure S3). The networks were labeled SS (stringent physical interactions; stringent essentiality), ST (stringent physical interactions; tolerant essentiality), TS (tolerant physical interactions; stringent essentiality) and TT (tolerant physical interactions; tolerant essentiality). Additionally, we built two further control networks to take into account that affinity capture methods could identify members of the same complex not having a straight physical interaction. Starting from the strictest network (SS), 1) we removed from the analysis all physical interactions that had only been found through affinity capture methods, resulting in a network containing 3811 edges; and, 2) we removed from the network any physical interaction that had not been detected by at least two different non-affinity capture methods, getting a network having 1762 edges.

### Random Models

All the analyses were compared to a null hypothesis of the emergence of essentiality or synthetic lethality from purely random events. The pool of genes was the whole list of yeast genes, except for those that we had previously removed from the analysis (essential genes participating in synthetic-lethality pairs through hypomorphic mutations). Moreover, we ensured that the random networks had no essentiality ambiguity by excluding from the randomisation the fixed-state genes; i.e. when creating random lists of essential genes, we excluded the members of synthetic-lethal pairs; when generating random synthetic-lethal pairs, we did not use essential genes. For each criterion, we generated 10000 random lists of genes to be assigned as essential genes and 10000 lists of pairs of proteins to be assigned as synthetic-lethal pairs. The lists contained the same number of genes (and pairs) as the original list. These lists were subsequently used to re-annotate the original network of physical interactions (see Figure S3). P-values were calculated as the number of random models having a result equal or higher as that obtained using the real data.

Being a network itself, the randomisation of the lists of synthetic lethal genes poses an additional problem: we cannot be sure if the observed differences are due to the identity of the nodes (the genes involved in synthetic lethality) or to changes in the topology of the network (see Table S20). As a further control we added two additional random models of synthetic lethality with the same degree distribution as the real network. Thus, these additional models have the same topology as the original synthetic-lethality network. In one model, we substituted each node in the synthetic lethality network with a gene from the whole pool of genes. In the other model we just used the genes already present in the synthetic lethality network and reshuffled the nodes. In the latter model the only difference between networks is the position of nodes within them, whereas in the former model each network can contain different nodes despite keeping the original topology (see Table S20). In both cases, we built 10000 lists, which were used to re-annotate the original network. P-values were calculated as above-mentioned.

### Random Exclusion

We randomly excluded some synthetic lethal pairs from the analyses. We used two different protocols: 1) excluding the same number of genes (regardless of the number of pairs) as in the real data (Model 1); and 2) excluding the same number of pairs (regardless of the number of genes) as in the real data (Model 2). We ran 10000 simulations for each analysis. P-values were calculated as above.

## Supporting Information

Figure S1 **Flowchart for selection of data of physical interactions.** We discarded data with just one evidence on the BioGRID database. Physical interactions detected using two different methods and reported at least twice independently were selected using the stringent criterion. The rest of physical interactions were selected through the tolerant criterion. All the interactions selected using the stringent criterion were also included in dataset obtained using the tolerant criterion.
(PPTX)

Figure S2 **Flowchart for selection of data of essentiality relationships (gene essentiality and synthetic lethality).** All synthetic lethality interactions present in the BioGRID database were selected using the tolerant criterion. However, only those with multiple evidences were selected using the stringent criterion. In the case of gene essentiality, we selected all the genes present in at least 3 datasets if using the tolerant criterion, and selected only those present in all datasets if using the stringent criterion. We did not use in our analyses data leading to ambiguity: essential genes involved in synthetic lethality interactions (probably as a result of a hypomorphic mutation), and their corresponding synthetic lethal pairs. Obviously, the dataset selected using the stringent criterion is a subset of the data selected using the tolerant criterion.
(PPTX)

Figure S3 **Annotation of physical interaction network with information on “essentiality”.**
(PPTX)

Table S1 **Analysis of paralogy of essential genes and members of synthetic-lethal pairs.**
(DOCX)

Table S2 **Analysis of function similarity of synthetic-lethal pairs.**
(DOCX)

Table S3 **Control for the different features of synthetic lethal and synthetic sick pairs.** P-values are calculated comparing the proportions obtained with the control and that of the synthetic lethal pairs selected using the stringent criteria and assuming a binomial distribution.
(DOCX)

Table S4 **Number of synthetic lethal pairs and their functional relatedness from the main contributing sources.** On the top, all pairs selected using the tolerant criterion are considered. On the bottom, only the pairs reported in a single study are taken into account.
(DOCX)

Table S5 **Percentage of synthetic-lethal pairs sharing at least one interactor.**
(DOCX)

Table S6 **Percentage of physical interactions occurring between two essential proteins.**
(DOCX)

Table S7 **Percentage of physical interactions occurring between members of synthetic-lethal pairs (it includes within and between pairs).**
(DOCX)

Table S8 **Percentage of physical interactions occurring between one member of a synthetic-lethal pair and an essential protein.**
(DOCX)

Table S9 **Analysis of the effect of affinity capture methods on the detection of physical interactions involving essential genes or members of synthetic lethal pairs in the SS network.** P-values are calculated comparing the proportions obtained with the control and that of the original network and assuming a binomial distribution.
(DOCX)

Table S10 **Analysis of the effect of self-interaction upon the essential subnetwork.** P-values are calculated comparing the proportions obtained with the control and that of the original network and assuming a binomial distribution.
(DOCX)

Table S11 **Analysis of essentiality on transient and obligate interaction networks (essential subnetwork).** P-values are calculated comparing both proportions and assuming a binomial distribution.
(DOCX)

Table S12 **Analysis of essentiality on transient and obligate interaction networks (interactions between products of single-essentiality genes).** P-values are calculated comparing both proportions and assuming a binomial distribution.
(DOCX)

Table S13 **Analysis of essentiality on transient and obligate interaction networks (interactions between members of synthetic lethal pairs).** P-values are calculated comparing both proportions and assuming a binomial distribution.
(DOCX)

Table S14 **Composition of transient and obligate physical interaction networks.** The first figure corresponds to the proportion of proteins within the transient network. The second figure corresponds to the proportion of proteins within the obligate network. P-values are calculated comparing both proportions and assuming a binomial distribution.
(DOCX)

Table S15 **Analysis of the importance of evolutionary and functional factors for the essential interactome.**
(DOCX)

Table S16 **Description of the filtering for selecting datasets of physical interactions.**
(DOCX)

Table S17 **Sets of methodologies most commonly used to detect physical interactions selected using the stringent criterion.**
(DOCX)

Table S18 **Description of the filtering and origin of synthetic-lethal interactions.** We considered high-throughput experiments those reporting more than 50 interactions, small-scale experiments those reporting five or less interactions, and medium-scale experiments those reporting between 6 and 50 interactions.
(DOCX)

Table S19 **Papers reporting multiple synthetic-lethal interactions selected using the stringent criterion.**
(DOCX)

Table S20 **Features of the randomized networks of synthetic lethal interactions.**
(DOCX)
