# Supplementary material for: The Role of Protein Interactions in Mediating Essentiality and Synthetic Lethality
Source: PLoS One. 2013 Apr 29;8(4):e62866. doi: 10.1371/journal.pone.0062866 (PMC3639263; doi:10.1371/journal.pone.0062866)
Supplement: Figure S1 — Flowchart for selection of data of physical interactions. We discarded data with just one evidence on the BioGRID database. Physical interactions detected using two different methods and reported at least twice independently were selected using the stringent criterion. The rest of physical interactions were selected through the tolerant criterion. All the interactions selected using the stringent criterion were also included in dataset obtained using the tolerant criterion. (PPTX) [file pone.0062866.s001.pptx]

## Slide 1
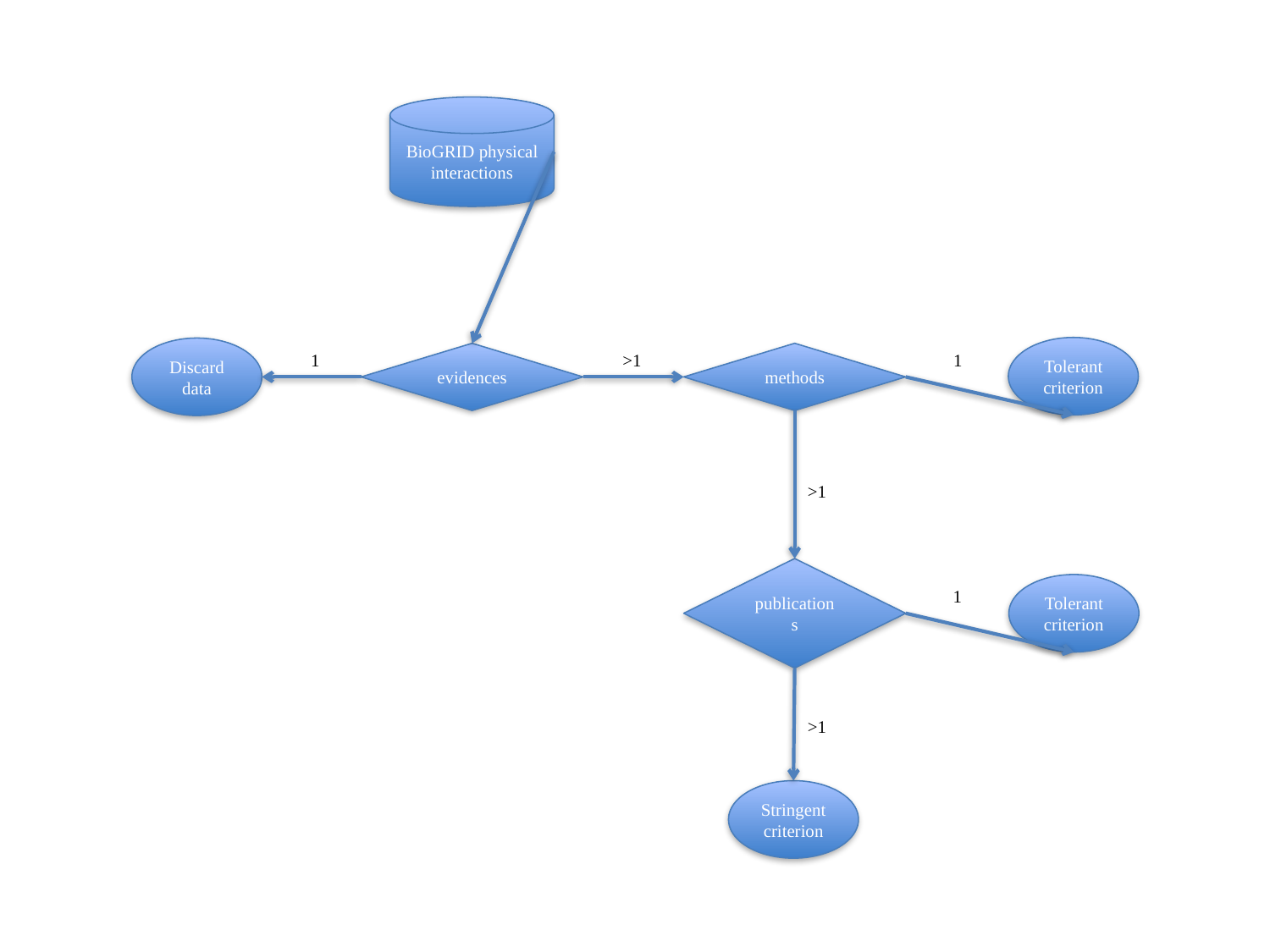

BioGRID physical interactions
evidences
methods
Tolerant criterion
Discard data
1
>1
1
>1
publications
Tolerant criterion
1
>1
Stringent criterion
