# Supplementary material for: The Role of Protein Interactions in Mediating Essentiality and Synthetic Lethality
Source: PLoS One. 2013 Apr 29;8(4):e62866. doi: 10.1371/journal.pone.0062866 (PMC3639263; doi:10.1371/journal.pone.0062866)
Supplement: Figure S2 — Flowchart for selection of data of essentiality relationships (gene essentiality and synthetic lethality). All synthetic lethality interactions present in the BioGRID database were selected using the tolerant criterion. However, only those with multiple evidences were selected using the stringent criterion. In the case of gene essentiality, we selected all the genes present in at least 3 datasets if using the tolerant criterion, and selected only those present in all datasets if using the stringent criterion. We did not use in our analyses data leading to ambiguity: essential genes involved in synthetic lethality interactions (probably as a result of a hypomorphic mutation), and their corresponding synthetic lethal pairs. Obviously, the dataset selected using the stringent criterion is a subset of the data selected using the tolerant criterion. (PPTX) [file pone.0062866.s002.pptx]

## Slide 1
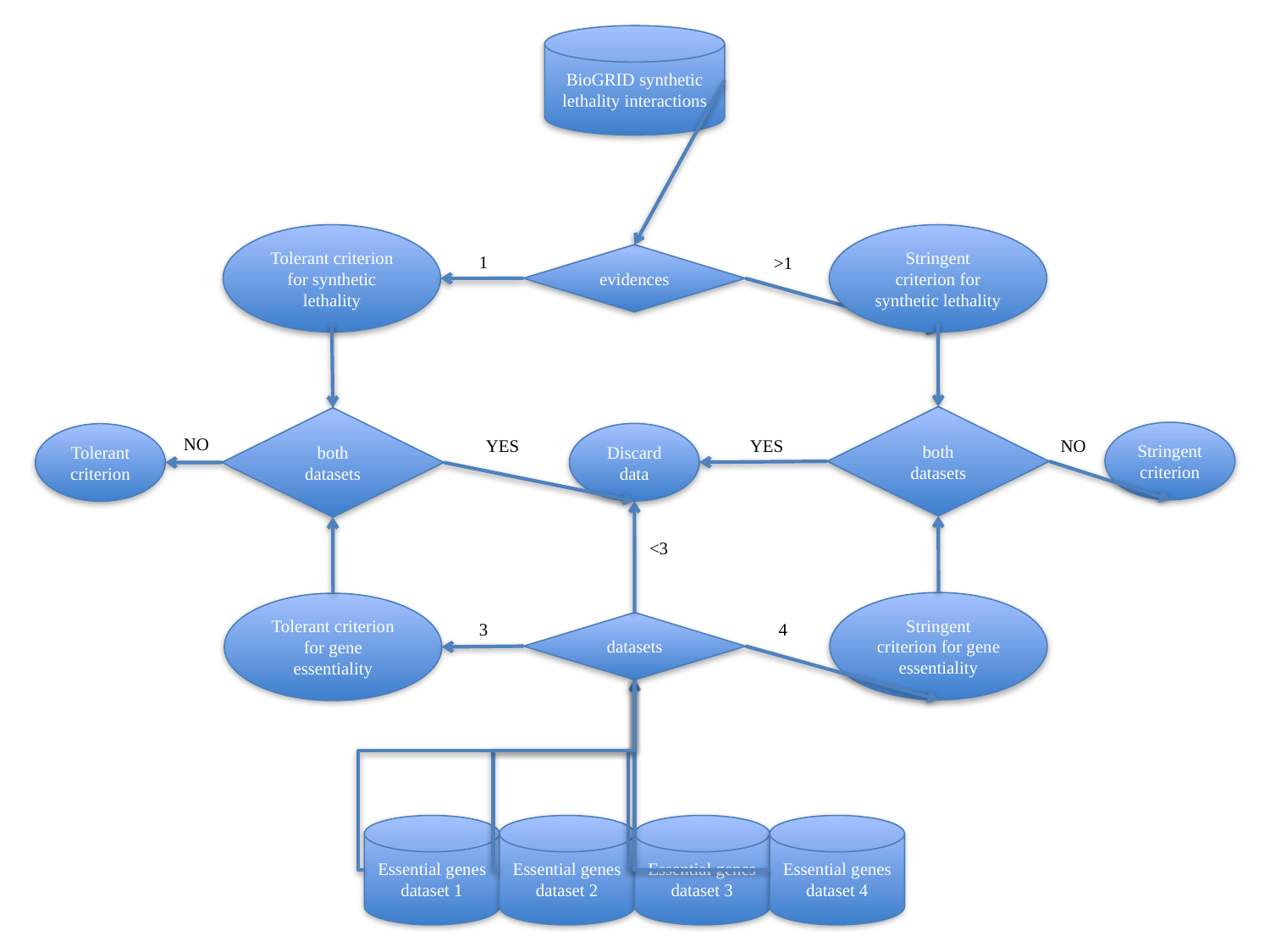

BioGRID synthetic lethality interactions
evidences
Stringent criterion for synthetic lethality
Tolerant criterion for synthetic lethality
1
>1
both datasets
both datasets
Stringent criterion
Discard data
Tolerant criterion
NO
NO
YES
YES
<3
datasets
Stringent criterion for gene essentiality
Tolerant criterion for gene essentiality
4
3
Essential genes dataset 2
Essential genes dataset 3
Essential genes dataset 1
Essential genes dataset 4
