# Supplementary material for: The Role of Protein Interactions in Mediating Essentiality and Synthetic Lethality
Source: PLoS One. 2013 Apr 29;8(4):e62866. doi: 10.1371/journal.pone.0062866 (PMC3639263; doi:10.1371/journal.pone.0062866)
Supplement: Figure S3 — Annotation of physical interaction network with information on “essentiality”. (PPTX) [file pone.0062866.s003.pptx]

## Slide 1
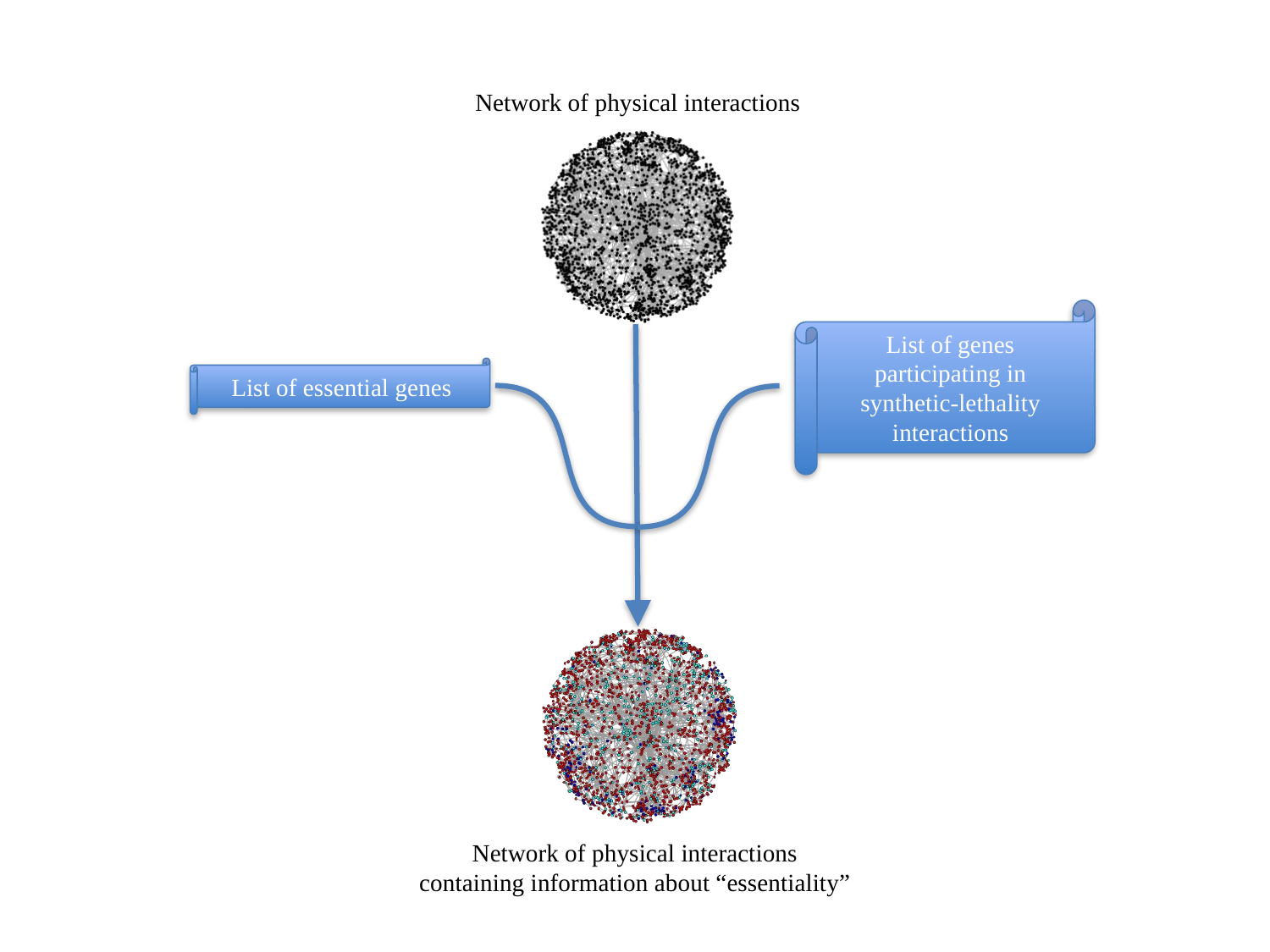

Network of physical interactions
List of genes participating in synthetic-lethality interactions
List of essential genes
Network of physical interactions
containing information about “essentiality”
