# Supplementary material for: The Role of Protein Interactions in Mediating Essentiality and Synthetic Lethality
Source: PLoS One. 2013 Apr 29;8(4):e62866. doi: 10.1371/journal.pone.0062866 (PMC3639263; doi:10.1371/journal.pone.0062866)
Supplement: Table S1 — Analysis of paralogy of essential genes and members of synthetic-lethal pairs. (DOCX) [file pone.0062866.s004.docx]

|  | **Stringent Criterion** | **Tolerant Criterion** |
| --- | --- | --- |
| **Essential genes that are singletons** | 79.1% (58.9±0.0%; p-value < 10^-4^) | 78.5% (62.5±0.0%; p-value < 10^-4^) |
| **Members of essential pairs that are singletons** | 50.1% (56.3±0.0%; p-value = 3*10^-4^) | 49.5% (56.1±0.0%; p-value < 10^-4^) |
| **Essential pairs containing two paralogues** | 4.3% (0.0±0.0%; p-value < 10^-4^) | 2.5% (0.0±0.0%; p-value < 10^-4^) |
