# Supplementary material for: The Role of Protein Interactions in Mediating Essentiality and Synthetic Lethality
Source: PLoS One. 2013 Apr 29;8(4):e62866. doi: 10.1371/journal.pone.0062866 (PMC3639263; doi:10.1371/journal.pone.0062866)
Supplement: Table S2 — Analysis of function similarity of synthetic-lethal pairs. (DOCX) [file pone.0062866.s005.docx]

|  | **Stringent Criterion** | **Tolerant Criterion** |
| --- | --- | --- |
| **Essential pairs with identical functional domain** | 5.4% (0.3±0.0%; p-value < 10^-4^) | 3.6% (0.2±0.0%; p-value < 10^-4^) |
| **Essential pairs with identical function assignment** | 60.0% (12.6±0.0%; p-value < 10^-4^) | 43.0% (12.4±0.0%; p-value < 10^-4^) |
