# Supplementary material for: The Role of Protein Interactions in Mediating Essentiality and Synthetic Lethality
Source: PLoS One. 2013 Apr 29;8(4):e62866. doi: 10.1371/journal.pone.0062866 (PMC3639263; doi:10.1371/journal.pone.0062866)
Supplement: Table S3 — Control for the different features of synthetic lethal and synthetic sick pairs. P-values are calculated comparing the proportions obtained with the control and that of the synthetic lethal pairs selected using the stringent criteria and assuming a binomial distribution. (DOCX) [file pone.0062866.s006.docx]

| **Control for Synthetic Lethality (N = 113)** |  |
| --- | --- |
| **Members of essential pairs that are singletons** | 50.9%; p- value ≈ 0.87 |
| **Essential pairs containing two paralogues** | 10.6%; p- value ≈ 0.03 |
| **Essential pairs with identical functional domain** | 8.8%; p- value ≈ 0.20 |
| **Essential pairs with identical function assignment** | 77.0%; p- value < 10^-4^ |
| **Control for Synthetic Sickness (N = 53902)** |  |
| **Members of essential pairs that are singletons** | 49.4%; p- value ≈ 0.55 |
| **Essential pairs containing two paralogues** | 0.4%; p- value < 10^-4^ |
| **Essential pairs with identical functional domain** | 0.9%; p- value < 10^-4^ |
| **Essential pairs with identical function assignment** | 24.0%; p- value < 10^-4^. |
