# Supplementary material for: The Role of Protein Interactions in Mediating Essentiality and Synthetic Lethality
Source: PLoS One. 2013 Apr 29;8(4):e62866. doi: 10.1371/journal.pone.0062866 (PMC3639263; doi:10.1371/journal.pone.0062866)
Supplement: Table S4 — Number of synthetic lethal pairs and their functional relatedness from the main contributing sources. On the top, all pairs selected using the tolerant criterion are considered. On the bottom, only the pairs reported in a single study are taken into account. (DOCX) [file pone.0062866.s007.docx]

| **Number of synthetic lethal pairs** | **Functional relatedness** | **Source** |
| --- | --- | --- |
| *All pairs* | | |
| 3129 | 40.0% | Tong et al. (2004) Science |
| 896 | 38.6% | Pan et al. (2006) Cell |
| 593 | 46.7% | Krogan et al. (2003) Mol Cell |
| 302 | 58.9% | Lin et al. (2008) Genes Dev |
| 272 | 21.0% | Zhao et al. (2005) Cell |
| 268 | 60.8% | Lesage et al. (2005) BMC Genet |
| 267 | 30.0% | Schoner et al. (2008) BMC Syst Biol |
| 252 | 56.7% | Deutscher et al. (2006) Nat Genet |
| 214 | 60.3% | Daniel et al. (2006) Genetics |
| 195 | 55.9% | Tong et al. (2001) Science |
| *Pairs only reported in a single study* | | |
| 2079 | 29.9% | Tong et al. (2004) Science |
| 722 | 35.0% | Pan et al. (2006) Cell |
| 281 | 47.3% | Krogan et al. (2003) Mol Cell |
| 271 | 21.0% | Zhao et al. (2005) Cell |
| 262 | 56.5% | Lin et al. (2008) Genes Dev |
| 245 | 25.7% | Schoner et al. (2008) BMC Syst Biol |
| 238 | 54.2% | Deutscher et al. (2006) Nat Genet |
| 149 | 46.3% | Daniel et al. (2006) Genetics |
| 91 | 36.3% | Tong et al. (2001) Science |
| 88 | 15.9% | Ye et al. (2005) Mol Ssyt Biol |
