# Supplementary material for: The Role of Protein Interactions in Mediating Essentiality and Synthetic Lethality
Source: PLoS One. 2013 Apr 29;8(4):e62866. doi: 10.1371/journal.pone.0062866 (PMC3639263; doi:10.1371/journal.pone.0062866)
Supplement: Table S5 — Percentage of synthetic-lethal pairs sharing at least one interactor. (DOCX) [file pone.0062866.s008.docx]

|  | **Stringent-Stringent** | **Stringent-Tolerant** | **Tolerant-Stringent** | **Tolerant-Tolerant** |
| --- | --- | --- | --- | --- |
| **Real network** | 7.6% | 4.7% | 14.3% | 9.8% |
| **Pure random network** | 0.1±0.0%; p-value < 10^-4^ | 0.1±0.0%; p-value < 10^-4^ | 0.6±0.0%; p-value < 10^-4^ | 0.5±0.0%; p-value < 10^-4^ |
| **Random network with fixed degree distribution (node substitution)** | 0.1±0.0%; p-value < 10^-4^ | 0.1±0.0%; p-value < 10^-4^ | 0.5±0.0%; p-value < 10^-4^ | 0.5±0.0%; p-value < 10^-4^ |
| **Random network with fixed nodes and degree distribution (node reshuffling)** | 0.7±0.0%; p-value < 10^-4^ | 0.2±0.0%; p-value < 10^-4^ | 3.1±0.0%; p-value < 10^-4^ | (1.5±0.0%; p-value < 10^-4^ |
