# Supplementary material for: The Role of Protein Interactions in Mediating Essentiality and Synthetic Lethality
Source: PLoS One. 2013 Apr 29;8(4):e62866. doi: 10.1371/journal.pone.0062866 (PMC3639263; doi:10.1371/journal.pone.0062866)
Supplement: Table S6 — Percentage of physical interactions occurring between two essential proteins. (DOCX) [file pone.0062866.s009.docx]

|  | **Network** | **Essential genes randomisation** |
| --- | --- | --- |
| **Stringent-Stringent** | 2.6% | 0.4±0.0%; p-value < 10^-4^ |
| **Stringent-Tolerant** | 3.1% | 0.3±0.0%; p-value < 10^-4^ |
| **Tolerant-Stringent** | 3.4% | 0.3±0.0%; p-value < 10^-4^ |
| **Tolerant-Tolerant** | 3.8% | 0.3±0.0%; p-value < 10^-4^ |
