# Supplementary material for: The Role of Protein Interactions in Mediating Essentiality and Synthetic Lethality
Source: PLoS One. 2013 Apr 29;8(4):e62866. doi: 10.1371/journal.pone.0062866 (PMC3639263; doi:10.1371/journal.pone.0062866)
Supplement: Table S7 — Percentage of physical interactions occurring between members of synthetic-lethal pairs (it includes within and between pairs). (DOCX) [file pone.0062866.s010.docx]

|  | **Network** | **Essential pairs randomisation** |
| --- | --- | --- |
| **Stringent-Stringent** | 9.7% | 1.2±0.0%; p-value < 10^-4^ |
| **Stringent-Tolerant** | 25.5% | 6.7±0.0%; p-value < 10^-4^ |
| **Tolerant-Stringent** | 6.9% | 1.0±0.0%; p-value < 10^-4^ |
| **Tolerant-Tolerant** | 20.6% | 6.4±0.0%; p-value < 10^-4^ |
