# Supplementary material for: The Role of Protein Interactions in Mediating Essentiality and Synthetic Lethality
Source: PLoS One. 2013 Apr 29;8(4):e62866. doi: 10.1371/journal.pone.0062866 (PMC3639263; doi:10.1371/journal.pone.0062866)
Supplement: Table S8 — Percentage of physical interactions occurring between one member of a synthetic-lethal pair and an essential protein. (DOCX) [file pone.0062866.s011.docx]

|  | **Network** | **Essential genes randomisation** | **Essential pairs randomisation** |
| --- | --- | --- | --- |
| **Stringent-Stringent** | 2.5% | 1.3±0.0%; p-value = 1*10^-4^ | 1.0±0.0%; p-value < 10^-4^ |
| **Stringent-Tolerant** | 5.3% | 1.8±0.0%; p-value < 10^-4^ | 2.7±0.0%; p-value < 10^-4^ |
| **Tolerant-Stringent** | 2.9% | 1.4±0.0%; p-value < 10^-4^ | 1.2±0.0%; p-value < 10^-4^ |
| **Tolerant-Tolerant** | 6.3% | 2.3±0.0%; p-value < 10^-4^ | 3.6±0.0%; p-value < 10^-4^ |
