# Supplementary material for: The Role of Protein Interactions in Mediating Essentiality and Synthetic Lethality
Source: PLoS One. 2013 Apr 29;8(4):e62866. doi: 10.1371/journal.pone.0062866 (PMC3639263; doi:10.1371/journal.pone.0062866)
Supplement: Table S9 — Analysis of the effect of affinity capture methods on the detection of physical interactions involving essential genes or members of synthetic lethal pairs in the SS network. P-values are calculated comparing the proportions obtained with the control and that of the original network and assuming a binomial distribution. (DOCX) [file pone.0062866.s012.docx]

| **Removal of physical interactions only detected through affinity capture methods** |  |
| --- | --- |
| **Percentage of physical interactions involving essential genes or members of synthetic lethal pairs** | 13.7%; p- value ≈ 0.20 |
| **Number of physical interaction occurring between two essential proteins** | 77 |
| **Number of physical interacting occurring between members of synthetic-lethal pairs** | 358 |
| **Number of physical interacting occurring between one member of a synthetic-lethal pair and an essential protein** | 89 |
| **Removal of physical interactions not detected through two different non-affinity capture methods** |  |
| **Percentage of physical interactions involving essential genes or members of synthetic lethal pairs** | 13.8%; p-value ≈ 0.37 |
| **Number of physical interaction occurring between two essential proteins** | 32 |
| **Number of physical interacting occurring between members of synthetic-lethal pairs** | 187 |
| **Number of physical interacting occurring between one member of a synthetic-lethal pair and an essential protein** | 25 |
