# Supplementary material for: The Role of Protein Interactions in Mediating Essentiality and Synthetic Lethality
Source: PLoS One. 2013 Apr 29;8(4):e62866. doi: 10.1371/journal.pone.0062866 (PMC3639263; doi:10.1371/journal.pone.0062866)
Supplement: Table S10 — Analysis of the effect of self-interaction upon the essential subnetwork. P-values are calculated comparing the proportions obtained with the control and that of the original network and assuming a binomial distribution. (DOCX) [file pone.0062866.s013.docx]

|  | Essential hetero-interactions | Homo-complexes involving essential genes | Homo-complexes involving members of synthetic pairs |
| --- | --- | --- | --- |
| **Stringent-Stringent** | 14.1% (N=4694); p-value ≈ 0.43 | 3.1% (N=257); p-value ≈ 0.63 | 21.8% (N=257); p- value < 10^-4^ |
| **Stringent-Tolerant** | 33.2% (N=4694); p-value ≈ 0.44 | 3.5% (N=257); p-value ≈ 0.74 | 44.0% (N=257); p- value < 10^-4^ |
| **Tolerant-Stringent** | 12.8% (N=11765); p-value ≈ 0.37 | 5.4% (N=388); p-value ≈ 0.08 | 19.6% (N=388); p- value < 10^-4^ |
| **Tolerant-Tolerant** | 30.2% (N=11765); p-value ≈ 0.32 | 5.9% (N=388); p-value ≈ 0.08 | 42.8% (N=388); p- value < 10^-4^ |
