# Supplementary material for: The Role of Protein Interactions in Mediating Essentiality and Synthetic Lethality
Source: PLoS One. 2013 Apr 29;8(4):e62866. doi: 10.1371/journal.pone.0062866 (PMC3639263; doi:10.1371/journal.pone.0062866)
Supplement: Table S11 — Analysis of essentiality on transient and obligate interaction networks (essential subnetwork). P-values are calculated comparing both proportions and assuming a binomial distribution. (DOCX) [file pone.0062866.s014.docx]

|  | **Transient interactions** | **Obligate interactions** | **P-value** |
| --- | --- | --- | --- |
| **Stringent-Stringent** | 17.0% (N=223) | 15.3% (N=2965) | ≈ 0.52 |
| **Stringent-Tolerant** | 40.4% (N=223) | 32.9% (N=2965) | < 0.05 |
| **Tolerant-Stringent** | 11.6% (N=717) | 15.4% (N=6740) | < 0.01 |
| **Tolerant-Tolerant** | 31.8% (N=717) | 30.8% (N=6740) | ≈ 0.58 |
