# Supplementary material for: The Role of Protein Interactions in Mediating Essentiality and Synthetic Lethality
Source: PLoS One. 2013 Apr 29;8(4):e62866. doi: 10.1371/journal.pone.0062866 (PMC3639263; doi:10.1371/journal.pone.0062866)
Supplement: Table S13 — Analysis of essentiality on transient and obligate interaction networks (interactions between members of synthetic lethal pairs). P-values are calculated comparing both proportions and assuming a binomial distribution. (DOCX) [file pone.0062866.s016.docx]

|  | **Transient interactions** | **Obligate interactions** | **P-value** |
| --- | --- | --- | --- |
| **Stringent-Stringent** | 13.9% (N=223) | 9.1% (N=2965) | < 0.05 |
| **Stringent-Tolerant** | 35.9% (N=223) | 22.7% (N=2965) | < 10^-4^ |
| **Tolerant-Stringent** | 9.5% (N=717) | 7.0% (N=6740) | < 0.05 |
| **Tolerant-Tolerant** | 27.9% (N=717) | 18.1% (N=6740) | < 10^-4^ |
