# Supplementary material for: The Role of Protein Interactions in Mediating Essentiality and Synthetic Lethality
Source: PLoS One. 2013 Apr 29;8(4):e62866. doi: 10.1371/journal.pone.0062866 (PMC3639263; doi:10.1371/journal.pone.0062866)
Supplement: Table S14 — Composition of transient and obligate physical interaction networks. The first figure corresponds to the proportion of proteins within the transient network. The second figure corresponds to the proportion of proteins within the obligate network. P-values are calculated comparing both proportions and assuming a binomial distribution. (DOCX) [file pone.0062866.s017.docx]

|  | **Number of essential genes** | **Number of members of synthetic lethal pairs** |
| --- | --- | --- |
| **Stringent-Stringent** | 4.0% \| 12.1%; p- value < 10^-4^ | 21.8% \| 22.2%; p-value ≈ 0.90 |
| **Stringent-Tolerant** | 4.8% \| 13.8%; p- value < 10^-4^ | 48.8% \| 42.7%; p-value ≈ 0.08 |
| **Tolerant-Stringent** | 3.0% \| 11.8%; p- value < 10^-4^ | 18.0% \| 20.6%; p-value ≈ 0.15 |
| **Tolerant-Tolerant** | 3.6% \| 13.4%; p- value < 10^-4^ | 44.2% \| 42.6%; p-value ≈ 0.48 |
