# Supplementary material for: The Role of Protein Interactions in Mediating Essentiality and Synthetic Lethality
Source: PLoS One. 2013 Apr 29;8(4):e62866. doi: 10.1371/journal.pone.0062866 (PMC3639263; doi:10.1371/journal.pone.0062866)
Supplement: Table S15 — Analysis of the importance of evolutionary and functional factors for the essential interactome. (DOCX) [file pone.0062866.s018.docx]

| **Protocol 1** | **Original Network** | **Exclusion of paralogues** | **Exclusion of pairs sharing interactor** |
| --- | --- | --- | --- |
| **Stringent-Stringent** | 14.7% | 12.8% (12.2±0.0%; p-value = 6*10^-3^) | 9.7% (10.1±0.0%; p-value = 0.1086) |
| **Stringent-Tolerant** | 34.0% | 30.0% (29.2±0.0%; p-value = 2.57 *10^-2^) | 22.4% (24.5±0.0%; p-value = 4*10^-4^) |
| **Tolerant-Stringent** | 13.2% | 11.6% (11.3±0.0%; p-value = 0.1082) | 8.3% (8.9±0.0%; p-value = 7.4*10^-3^) |
| **Tolerant-Tolerant** | 30.8% | 27.0% (26.9±0.0%; p-value = 0.3244) | 18.6% (21.7±0.0%; p-value < 10^-4^) |
| **Protocol 2** | **Original Network** | **Exclusion of paralogues** | **Exclusion of pairs sharing interactor** |
| **Stringent-Stringent** | 14.7% | 12.8% (12.3±0.0%; p-value = 1.45*10^-2^) | 9.7% (11.2±0.0%; p-value < 10^-4^) |
| **Stringent-Tolerant** | 34.0% | 30.0% (27.7±0.0%; p-value < 10^-4^) | 22.4% (24.7±0.0%; p-value = 2*10^-4^) |
| **Tolerant-Stringent** | 13.2% | 11.6% (11.5±0.0%; p-value = 0.3305) | 8.3% (9.5±0.0%; p-value < 10^-4^) |
| **Tolerant-Tolerant** | 30.8% | 27.0% (25.9±0.0%; p-value = 3*10^-4^) | 18.6% (20.0±0.0%; p-value = 1.5*10^-3^) |
