# Supplementary material for: The Role of Protein Interactions in Mediating Essentiality and Synthetic Lethality
Source: PLoS One. 2013 Apr 29;8(4):e62866. doi: 10.1371/journal.pone.0062866 (PMC3639263; doi:10.1371/journal.pone.0062866)
Supplement: Table S16 — Description of the filtering for selecting datasets of physical interactions. (DOCX) [file pone.0062866.s019.docx]

| Number of non-redundant physical interactions in the BioGRID repository | 48087 |
| --- | --- |
| Number of physical interactions with at least two pieces of evidence of being true PPIs (tolerant criterion) | 12153 |
| Number of physical interactions reported in at least two publications | 9983 |
| Number of physical interactions identified using at least two different techniques | 5805 |
| Number of physical interactions reported at least twice and identified using at least two techniques (stringent criterion) | 4951 |
