# Supplementary material for: The Role of Protein Interactions in Mediating Essentiality and Synthetic Lethality
Source: PLoS One. 2013 Apr 29;8(4):e62866. doi: 10.1371/journal.pone.0062866 (PMC3639263; doi:10.1371/journal.pone.0062866)
Supplement: Table S17 — Sets of methodologies most commonly used to detect physical interactions selected using the stringent criterion. (DOCX) [file pone.0062866.s020.docx]

| **Methodologies** | **Number of detected physical interactions** |
| --- | --- |
| Affinity Capture-MS  Affinity Capture-Western | 1134 |
| Affinity Capture-MS  Two-hybrid | 361 |
| Affinity Capture-MS  Affinity Capture-Western  Two-hybrid | 243 |
| Affinity Capture-Western  Two-hybrid | 240 |
| Affinity Capture-MS  Affinity Capture-Western  Co-purification | 218 |
| Affinity Capture-MS  Co-purification | 139 |
| Affinity Capture-MS  Affinity Capture-Western  Reconstituted Complex | 126 |
| Affinity Capture-Western  Reconstituted Complex  Two-hybrid | 119 |
| Affinity Capture-MS  PCA | 115 |
| Affinity Capture-Western  Reconstituted Complex | 111 |
| Reconstituted Complex  Two-hybrid | 101 |
| Affinity Capture-MS  Affinity Capture-Western  Reconstituted Complex  Two-hybrid | 97 |
| PCA  Two-hybrid | 95 |
| Affinity Capture-MS  Reconstituted Complex | 75 |
| Affinity Capture-MS  Affinity Capture-Western  Co-purification  Two-hybrid | 57 |
| Affinity Capture-MS  Affinity Capture-Western  Co-purification  Reconstituted Complex | 53 |
| Affinity Capture-Western  Co-purification | 51 |
| Affinity Capture-MS  Affinity Capture-Western  Co-fractionation | 47 |
| Affinity Capture-MS  Affinity Capture-Western  PCA | 41 |
| Affinity Capture-MS  Reconstituted Complex  Two-hybrid | 41 |
