# Supplementary material for: The Role of Protein Interactions in Mediating Essentiality and Synthetic Lethality
Source: PLoS One. 2013 Apr 29;8(4):e62866. doi: 10.1371/journal.pone.0062866 (PMC3639263; doi:10.1371/journal.pone.0062866)
Supplement: Table S18 — Description of the filtering and origin of synthetic-lethal interactions. We considered high-throughput experiments those reporting more than 50 interactions, small-scale experiments those reporting five or less interactions, and medium-scale experiments those reporting between 6 and 50 interactions. (DOCX) [file pone.0062866.s021.docx]

| Number of non-redundant synthetic-lethal interactions in the BioGRID repository | 12021 |
| --- | --- |
| Number of synthetic-lethal interactions after exclusion of interactions containing essential genes (tolerant criterion) | 8055 |
| Detected in one or multiple high-throughput experiments | 6008 |
| Detected in one or multiple medium-scale experiments | 754 |
| Detected in one or multiple small-scale experiments | 771 |
| Detected in multiple experiments of different scale | 522 |
| Number of synthetic-lethal interactions reported at least twice after exclusion of interactions containing essential genes (tolerant criterion) | 1621 |
| Detected in multiple high-throughput experiments | 977 |
| Detected in multiple medium-scale experiments | 15 |
| Detected in multiple small-scale experiments | 96 |
| Detected in multiple experiments of different scale | 533 |
