# Supplementary material for: The Role of Protein Interactions in Mediating Essentiality and Synthetic Lethality
Source: PLoS One. 2013 Apr 29;8(4):e62866. doi: 10.1371/journal.pone.0062866 (PMC3639263; doi:10.1371/journal.pone.0062866)
Supplement: Table S19 — Papers reporting multiple synthetic-lethal interactions selected using the stringent criterion. (DOCX) [file pone.0062866.s022.docx]

| **Papers reporting interactions** | **Number of interactions** |
| --- | --- |
| Tong et al. (2004) Science  Lesage et al. (2005) BMC Genet | 228 |
| Krogan et al. (2003) Mol Cell  Tong et al. (2004) Science | 130 |
| Tong et al. (2004) Science  Lesage et al. (2004) Genetics | 116 |
| Tong et al. (2004) Science  Friesen et al. (2006) Mol Biol Cell | 76 |
| Krogan et al. (2003) Mol Cell  Krogan et al. (2004) PNAS | 53 |
| Tong et al. (2004) Science  Pan et al. (2004) Mol Cell | 37 |
| Tong et al. (2004) Science  Daniel et al. (2006) Genetics | 30 |
| Krogan et al. (2003) Mol Cell Biol  Krogan et al. (2003) Mol Cell | 29 |
| Tong et al. (2004) Science  Lesage et al. (2004) Genetics  Durand et al. (2008) Yeast | 26 |
| Tong et al. (2004) Science  Pan et al. (2006) Cell | 24 |
