# Supplementary material for: The Role of Protein Interactions in Mediating Essentiality and Synthetic Lethality
Source: PLoS One. 2013 Apr 29;8(4):e62866. doi: 10.1371/journal.pone.0062866 (PMC3639263; doi:10.1371/journal.pone.0062866)
Supplement: Table S20 — Features of the randomized networks of synthetic lethal interactions. (DOCX) [file pone.0062866.s023.docx]

| **Strategy** | **Topology** | **Number of nodes** | **Identity of nodes** | **Number of edges** |
| --- | --- | --- | --- | --- |
| **Pure random construction** | Different for each randomization | As original network | Different for each randomization | As original network |
| **Nodes substitution** | As original network | As original network | Different for each randomization | As original network |
| **Nodes shuffling** | As original network | As original network | As original network | As original network |
